# Design of Linear and Cyclic Mutant Analogues of Dirucotide Peptide (MBP_82–98_) against Multiple Sclerosis: Conformational and Binding Studies to MHC Class II

**DOI:** 10.3390/brainsci8120213

**Published:** 2018-12-04

**Authors:** George Deraos, Eftichia Kritsi, Minos-Timotheos Matsoukas, Konstantina Christopoulou, Hubert Kalbacher, Panagiotis Zoumpoulakis, Vasso Apostolopoulos, John Matsoukas

**Affiliations:** 1 Department of Chemistry, University of Patras, 26500 Patras, Greece; deraos@gmail.com (G.D.); christopouloucon@gmail.com (K.C.); 2 ELDrug S.A., Patras Science Park, Platani, 26504 Patras, Greece; 3 Institute of Biology, Medicinal Chemistry and Biotechnology, National Hellenic Research Foundation, 11635 Athens, Greece; ekritsi@eie.gr; 4 Department of Pharmacy, University of Patras, 26500 Patras, Greece; minmatsoukas@gmail.com; 5 Interfaculty Institute of Biochemistry, University of Tubingen, 72076 Tubingen, Germany; kalbacher@uni-tuebingen.de; 6 Institute for Health and Sport, Victoria University, Melbourne VIC 3030, Australia

**Keywords:** multiple sclerosis, MS, cyclic peptide, MBP, dirucotide, HLA-DR2, HLA-DR4, myelin basic protein, altered peptide ligand

## Abstract

Background: Multiple sclerosis (MS) is an autoimmune disorder of the central nervous system. MS is a T cell-mediated disease characterized by the proliferation, infiltration, and attack of the myelin sheath by immune cells. Previous studies have shown that cyclization provides molecules with strict conformation that could modulate the immune system. Methods: In this study, we synthesized peptide analogues derived from the myelin basic protein (MBP)_82–98_ encephalitogenic sequence (dirucotide), the linear altered peptide ligand MBP_82–98_ (Ala^91^), and their cyclic counterparts. Results: The synthesized peptides were evaluated for their binding to human leukocyte antigen (HLA)-DR2 and HLA-DR4 alleles, with cyclic MBP_82–98_ being a strong binder with the HLA-DR2 allele and having lower affinity binding to the HLA-DR4 allele. In a further step, conformational analyses were performed using NMR spectroscopy in solution to describe the conformational space occupied by the functional amino acids of both linear and cyclic peptide analogues. This structural data, in combination with crystallographic data, were used to study the molecular basis of their interaction with HLA-DR2 and HLA-DR4 alleles. Conclusion: The cyclic and APL analogues of dirucotide are promising leads that should be further evaluated for their ability to alter T cell responses for therapeutic benefit against MS.

## 1. Introduction

Multiple sclerosis (MS) is a chronic neurodegenerative disease that affects protein constituents within the myelin sheath, such as myelin basic protein (MBP), myelin oligodendrocyte glycoprotein, and proteolipid protein. One of the characteristics of MS is the presence of encephalitogenic CD4^+^ T helper (h) 1 cells mediating disease, which recognize peptide epitopes such as MBP_83–99_, MBP_87–99_, and MBP_1–22_. These agonist peptide epitopes and their altered peptide ligands (APL; mutation by one to two amino acids) have been extensively studied by our group and others for their ability to modulate immune responses [1,2,3,4,5,6,7,8,9,10] and/or inhibit experimental autoimmune encephalomyelitis (EAE), which is one of the best animal models to study MS. An association of MS with the human leukocyte antigen (HLA)-class II is well established, linking MS to HLA-DR2 (DRB1*1501) and HLA-DR4 (DRB1*0401) alleles [11]. HLA-DR2 and HLA-DR4 are involved in the presentation of myelin epitopes to their surface on antigen-presenting cells to the T cell receptor (TCR).

Cyclic peptides are considered as mimetic candidates of their linear relatives and as modulators of immune cells. Cyclic peptides pose a number of advantages compared to their linear counterparts [12] in that they are more resistant to degradation by proteolytic enzymes [2,9,10,13,14]. In addition, cyclic peptides are conformationally restricted, and may constitute a key step toward the design and development of non-peptide mimetics. Previously, we reported that the APL of MBP_87–99_ with one to two amino acid modifications at the principal TCR contact sites diverted immune response from Th1 to Th2 and generated antibodies that did not cross-react with the native MBP protein [5,6,7]. In addition, cyclic APLs, including cyclo (91–99) (Ala^96^) MBP_87–99_ and cyclo (87–99) (Arg^91^, Ala^96^) MBP_87–99_, have a significant impact on the proliferation and cytokine profile of MBP_87–99_-specific CD4^+^ T cells from MS patients. Such cyclic APL were also shown to bind to HLA-DR4, and were more stable to lysosomal enzymes than their linear counterparts [2,9,10]. These findings are a preliminary step toward the design and synthesis of linear and cyclic APLs as promising candidates that may affect antigen presentation to the TCR and regulate immune response.

The 17-mer linear peptide MBP_82–98_ (known as dirucotide; DENPVVHFF**K**^91^NIVTPRT) was shown to suppress autoreactive T cells following intravenous injections in preclinical and clinical studies [15]. In a phase II human clinical trial in HLA-DR2 and HLA-DR4 patients with MS, dirucotide was shown to be safe and prolonged the median time to disease progression [16]. Dirucotide is licensed to BioMS Medical Corp and shared the development with Eli Lilly and Company. In a phase III clinical trial of 612 subjects, there were no enhanced primary and secondary endpoints achieved. Thus, in the population studied, dirucotide did not provide any benefit compared to placebo [17]. As a consequence, all further clinical trials were discontinued. However, it was later shown that at six weeks and six months after treatment, there was an increase in the number of CD4^+^CD25^+^Foxp3^+^ regulatory T cells; hence, there is the possibility that dirucotide is capable of reversing peripheral anergy and inducing T cell regulation [18]. In an attempt to improve the actions of dirucotide and re-establish its potential to induce regulatory T cells, we mutated position 91 to Ala (TCR contact residue; DENPVVHFF**A**^91^NIVTPRT) to result in the APL, linear MBP_82–98_ (Ala^91^). In addition, we cyclized dirucotide and its APL counterpart in order to improve its stability. We describe their synthesis, binding affinity to HLA-DR2 and HLA-DR4 alleles, and conformational analysis by nuclear magnetic resonance (NMR) and molecular dynamics simulations in complex with HLA-DR2 and HLA-DR4. We confirm that cyclic peptides bind to HLA-DR2 and HLA-DR4, which are peptides that have a higher stability to degradation compared to their linear counterpart [9], opening new avenues for the discovery of enhanced new generation immunomodulators in the treatment of MS. These studies pave the way for linear and cyclic dirucotide mutants to be conjugated to mannan, in order to further regulate immune responses [2,3,4,7,19].

## 2. Materials and Methods

### 2.1. Chemistry

#### 2.1.1. Solid-Phase Peptide Synthesis of Linear and Cyclic-MBP_82–98_ Analogues

All peptide analogues of MBP_83–99_ and MBP_82–98_ (DENPVVHFFKNIVTPRT), as shown in Table 1, were synthesized in solid phase using 2-chlorotrityl chloride resin (CTLR-Cl, Barlos resin). The Fmoc/tBu conventional methodology was chosen as the best way for the synthesis of the peptide analogues, as previously described by our group [20]. All of the amino acids were purchased from CBL (Patras, Greece). The couplings were accomplished within three hours in order to reduce racemization and side effects during this process, which was controlled by chloranil test and thin-layer chromatography (TLC). For the Fmoc group, peptide removal was treated in every cycle with 25% piperidine in dimethylformamide (DMF) solution. The cleavage of the peptide from the resin using mild conditions of dichloromethane/trifluoroethyl alcohol (DCM/TFE), in a 7/3 solution, allowed the high yield of free from the resin protected peptide, and subsequently its head to tail cyclization.

#### 2.1.2. Cyclization Procedure

**Scheme 1:**
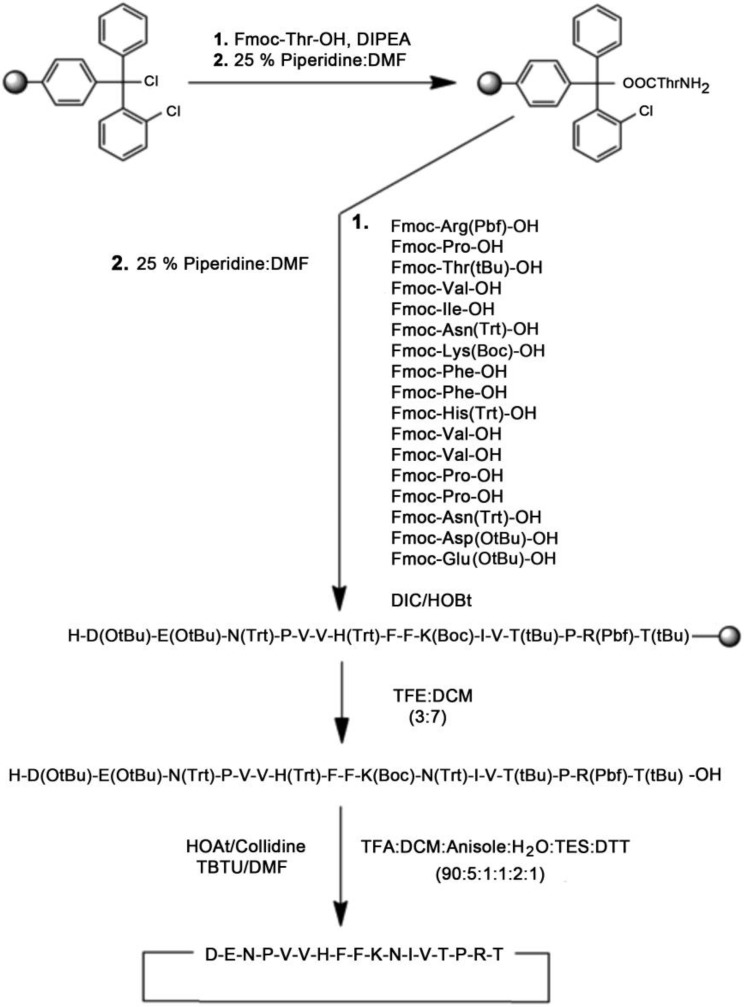
Schematic representation of the solid-phase peptide synthesis of a cyclic MBP_82–98_ peptide.

**Figure 1:**
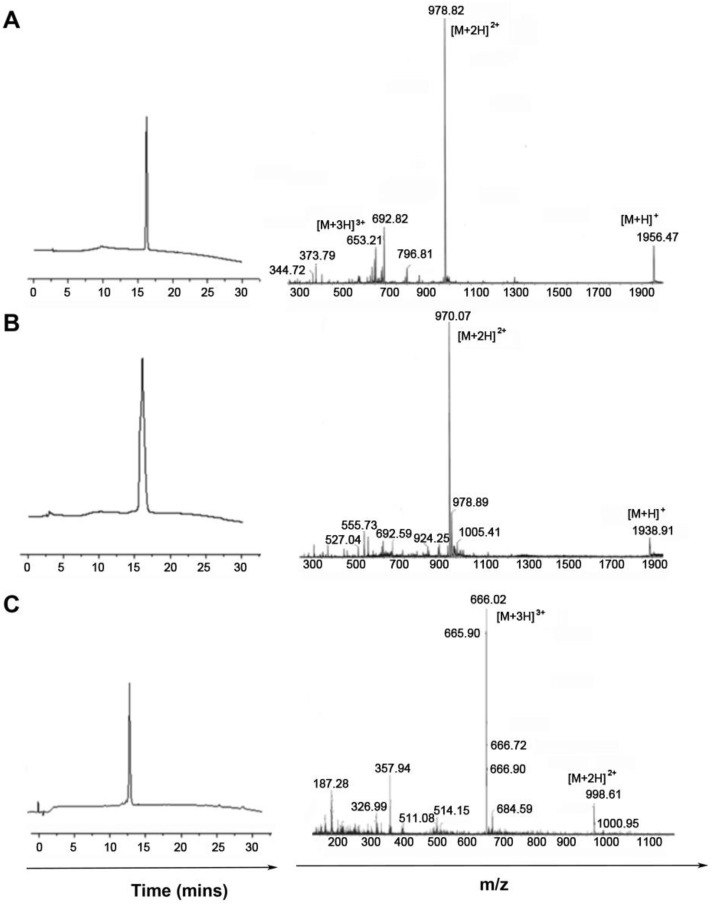
Analytical HPLC (left) and electron spray ionization mass spectrometry (ESI-MS) (right) of purified lyophilized (**A**) linear MBP_82–98_ (Ala^91^) peptide, (**B**) cyclic MBP_82–98_ (Ala^91^) peptide and (**C**) cyclic MBP_82–98_ peptide (solvent A: 0.1% trifluoroacetic acid in H_2_O, solvent B: 0.1% trifluoroacetic acid in ACN). Column Nucleosil RP-C18 RP, seven µm.

Cyclization of the linear protected MBP_82–98_ peptide was carried out in liquid phase. O-benzotriazol-1-yl-N,N,N’,N’-tetramethyluronium tetrafluoroborate, 1-hydroxy-7-azabenzotriazole, and 2,4,6 collidine were used as coupling reagents, allowing fast reactions and high yield of the cyclic protected analogue (Scheme 1) [20]. The mixture was stirred overnight, and the cyclization procedure was accomplished within 24 h and monitored by thin-layer chromatography using nbutanol/acetic acid/water (4/1/1) as the solvent system, and analytical HPLC using a 3.5-μm C4 Nucleosil RP column. The protected cyclic product was then treated with 95% trifluoroacetic acid in DCM solution containing 5% dithiothreitol/water/anisole/trimethyl saline as scavengers, leading to the final, free from side chain protected group’s cyclic peptide. Purification of the crude peptide analogues was performed under preparative HPLC using a seven-µm Nucleosil RP-C18 RP semi-preparative column (Figure 1).

### 2.2. Competition Binding of Linear and Cyclic MBP_82–98_ to HLA-DR2 and HLA-DR4

The binding of peptides to HLA-DR2 and HLA-DR4 was performed as previously demonstrated [21,22,23]. Briefly, epstein-barr virus transformed homozygous human B cell lines HTC-Lan (DRB1*1501, DRB5*0101) or BSM (DRB1*0401) were used for isolating MHC class II molecules. HTC-Lan and BSM cell pellets were lyzed by Nonidet P-40, and HLA-DR2/DR4 were isolated from homogenates by affinity chromatography using the monoclonal antibody L243. The purity of the preparation was checked by 12% SDS-PAGE (shown), high pressure size exclusion chromatography and western blotting (not shown). Competition binding assays for MBP_82–98_ peptide analogues were carried out using fluorescent 7-amino-4-methylcoumarin-3-acetic acid (AMCA)-labeled allele-specific MBP_83–99_ for HLA-DR2b chain or HA_306–318_-peptide for HLA-DR4. Solubilized HLA-DR2 (0.66 mM) and HLA-DR4 (0.11 mM) were incubated for 48 h at 37 °C with MBP_83–99_-(AMCA)-labeled peptide or N-terminally 7-amino-4-methylcoumarin-3-acetic acid (AMCA)-labeled influenza matrix peptide (AMCA-HA_306–318_-peptide), dissolved in 150 mM of sodium phosphate, pH 6.0, containing 15% acetonitrile, and 0.1% Zwittergent-12 (Calbiochem, San Diego, CA, USA). Competition assays were performed in a one-μM solution of AMCA peptide. All of the samples were analyzed on a Pharmacia Superdex 75 HR 5/20 high performance gel filtration column. The column was operated at a flow rate of 0.3–0.4 mL/min using the HPSEC buffer, pH 6.0. The eluent passed through a Shimadzu fluorescence spectrometer (350/450 nm) and a Merck ultraviolet detector (214 nm) set in series. Fluorescence and UV signals eluting with the HLA-DR dimers were recorded by a model D 2500 integrator (Merck-Hitachi). To determine the binding differences between 50-fold, 20-fold, and 10-fold excess of peptide binding to HLA-DR2 or HLA-DR4, GraphPad Prism (Version 7, GraphPad Software Inc, San Diego, CA, USA) was used to perform two-way ANOVA followed by Tukey’s multiple comparisons test to determine the significance between each dose, with *p* < 0.05 considered significant.

### 2.3. Structure Elucidation and Conformational Studies

#### 2.3.1. NMR Spectroscopy

For both cyclic and linear MBP_82–98_ (Ala^91^) analogues, data were acquired at 298 K on a Varian 600-MHz spectrometer equipped with a triple resonance probe. One-dimensional (1D) ^1^H-NMR, two-dimensional (2D) ^1^H-^1^H TOCSY, and 2D-^1^H-^1^H NOESY spectra were recorded using spectral width from −1 to 15 ppm without presaturation of the H_2_O signal. The pulse widths for all of the experiments were 6.25, and the number of increments was 256. The number of scans was set to 48 for the 2D ^1^H-^1^H TOCSY, and 56 for the 2D ^1^H-^1^H NOESY spectra. The mixing time was set t_m_ = 250, and the number of scans was equal to 56. For data processing and spectral analysis, Mnova software (version 6.2.0, Mestrelab Research S.L., Santiago de Compostela, Spain) was used.

A 2D TOCSY spectrum (total correlation spectroscopy) creates correlations between all of the protons within a given spin system, as long as there are couplings between every intervening proton. This is useful for identifying protons on amino acids. Magnetization may be transferred successively over up to five or six bonds as long as successive protons are coupled. Thus, all of the protons on a given amino acid show a correlation with all of the other protons on the same amino acid, but not with protons on different amino acids. A 2D NOESY spectrum is one of the most useful for conformational studies, since it allows correlating nuclei through space (at a distance smaller than 5Å). By measuring cross-peak intensity, distance information can be extracted.

#### 2.3.2. Conformational Analysis

A MacroModel module of Schrodinger 2013 package was used for the conformational studies. The linear and the cyclic analogues MBP_82–98_ (Ala^91^) were designed and minimized with an OPLS_2005 force field, the dielectric constant was set to 45, simulating the DMSO environment of the NMR solvent. Minimization was performed with the PRCG (Powell–Reeves conjugate gradient) algorithm, using 100,000 iterations and a convergence threshold of 0.01 kcal/mol Å. Energetically minimized conformations were further subjected to molecular dynamic simulations. During the molecular dynamics (MD) simulations, the method of stochastic dynamics was implemented; the simulation temperature was 600 K, the time step was equal to 1.5 fs, the equilibration time was equal to 2000 ps, and the simulation time was equal to 20,000 ps. Five hundred (500) conformations resulted after MD simulations, which were classified into 10 families (clusters), according to the rotation of dihedral angles φ (rotation: N-Ca), ψ (rotation: Ca-CO), and ω (rotation: CO-N). At a final stage, conformers that satisfy the distance constraints were minimized using the parameters mentioned above.

#### 2.3.3. Binding Mode of Peptide-HLA-DR Complexes

All of the crystal structures were visualized and studied using PyMOL (the PyMOL Molecular Graphics System, Version 1.8 Schrödinger, LLC., New York, NY, USA). Construction of the linear peptides was made by the direct replacement of amino acids in crystal structures of HLA-DR2b (Pdb code 1BX2) and HLA-DR4 (pdb code 4MCY) in PyMOL. Cyclization for MBP_82–98_ was performed manually by joining the C-terminal and N-terminal residues in Discovery Studio version 3.5 (Discovery Studio, version 3.5. Accelrys Inc; San Diego, CA, USA: 2012). Minimizations of all the HLA and all of the peptide analogues were done using the conjugate gradient algorithm for 500 steps in Discovery Studio version 3.5.

## 3. Results and Discussion

APL closely resemble their native (agonist) peptide, with one to two altered amino acid residues; also, usually for autoimmunity, they closely resemble those that interact with the TCR [24] as opposed to changes to residues within the peptide-binding groove, which is commonly altered for cancer peptides [25,26]. APL from dominant myelin epitopes in MS have shown promise in preclinical animal studies based on the effort to alter the cytokine profile (from Th1 to Th2) and/or induce regulatory T cells. Early studies showed that mutations to the peptide MBP_72–85_, which is an immunodominant epitope in Lewis rats, inhibited the stimulation of T cells and the clinical progression of EAE [27]. Likewise, the MBP_87–99_ immunodominant autoreactive T cell epitope, with one amino acid mutation at K^91^ to A^91^, also suppressed EAE in Lewis rats and reduced the secretion of pro-inflammatory cytokines, TNFα and IFNγ [28]. In addition, the longer agonist MBP_83–99_ peptide with one amino acid mutation ((Y^91^) MBP_83–99_) or two amino acid mutations ((R^91^, A^96^) MBP_83–99_) induced interleukin (IL)-4 secretion by T cells in mice, although IFNγ was still present [5]. When conjugated to reduced mannan, high IL-4 cytokines resulted, and no IFNγ (Th1 to Th2 switch) [6].

The cyclization of peptides is of great interest, since the partial stability of linear peptides restricts them as potential therapeutics. We have extensively studied cyclic peptides for their enhanced stability and their potential to be used as immunotherapeutics in MS due to their altered three-dimensional conformation. In fact, we recently showed that the cyclization of the immunodominant myelin oligodendrocyte glycoprotein (MOG_35–55_) T cell epitope induces mild transient acute EAE without chronic axonopathy [29]. Similarly, cyclization of the agonist proteolipid protein (PLP_139–151_) epitope resulted in low disease burden, and minimal inflammatory, demyelinating, and axonopathic pathology compared to the linear PLP_139–151_ peptide [30]. This data supported for the first time the concept that the cyclization of an established encephalitogenic agonist peptide ameliorates the underlying pathological processes of EAE, and provided promising new peptide leads for the immunotherapy of MS.

Dirucotide is a linear MBP_82–98_ peptide that, when injected in high concentrations in patients with MS, suppressed T cell responses against MBP_82–98_ epitope and delayed disease progression [16]. However, in larger phase III trials, no added benefit was noted compared to the placebo group in patients with relapsing remitting MS and in secondary progressive MS [17,18]. As a result, further planned/scheduled clinical trials were terminated. Of interest, dirucotide was shown to induce CD4^+^CD25^+^Foxp3^+^ regulatory T cells following its injection in patients with MS [18]. It is likely that the low stability of linear MBP_82–98_ peptide or that an agonist was used in these studies may have resulted in no additional benefit to patients in phase III trials. In an attempt to improve the outcomes of dirucotide, we designed and synthesized modified dirucotide peptides by either the APL approach (one amino acid mutation at position 81), or cyclization of the agonist dirucotide peptide. Here, we show their synthesis, their binding to HLA-DR2 and HLA-DR4, and their structural profile by NMR and molecular dynamics simulations in complex with HLA-DR2 and HLA-DR4.

### 3.1. Synthesis and Purification of Peptide Analogues

The purity of the final peptides was over 98%, as determined by HPLC analysis and electron spray ionization mass spectrometry (ESI-MS) (Figure 1).

### 3.2. Structure Elucidation and Conformational Studies of Cyclic and Linear Peptides

**Scheme 2:**
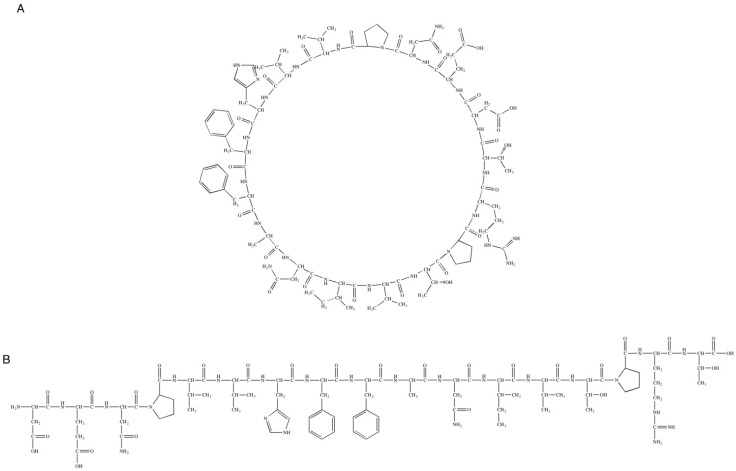
Chemical structure of (**A**) cyclic MBP_82–98_ (A^91^) and (**B**) linear MBP_82–98_ (A^91^).

**Figure 2:**
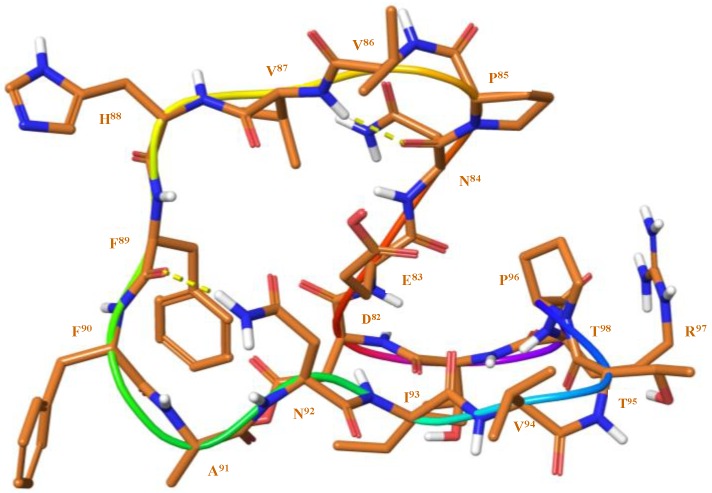
Low-energy conformer of cyclic MBP_82–98_ (A^91^) in accordance with the NOE distance constraints.

**Figure 3:**
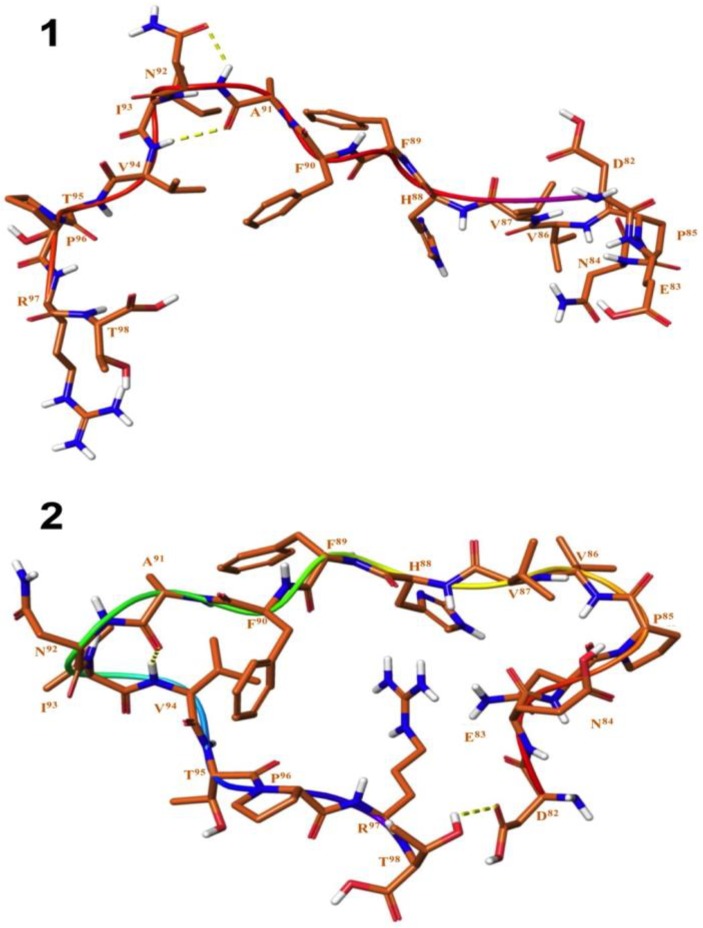
Low energy conformers of the linear MBP_82–98_ (A^91^), which satisfies the NOE distance constraints. Both demonstrate the interaction between A^91^ and V^94^, while the interaction between the terminal D^82^ and T^98^ is apparent for conformer 2.

The ^1^H NMR spectrum of cyclic MBP_82–98_ (A^91^) (Scheme 2A) is presented in Figure 2, and the NMR assignment is reported in Table 2. The crucial Nuclear Overhauser Effects (NOE) for the conformational arrangement of the peptide are presented in Table 3. Low-energy conformers of linear MBP_82–98_ (Ala^91^) that satisfy NOE distant constraints are shown in Figure 3.

The cyclic peptide was energetically minimized and subjected to molecular dynamics simulations. Produced conformers were classified into 10 different families (clusters), according to the rotation of dihedral angles φ (rotation: N-Ca), ψ (rotation: Ca-CO), and ω (rotation: CO-N). Low-energy conformers from each family were selected as representative ones. Representative conformers from all families are mainly characterized by a β-turn, following an (i, I + 3) interaction between the CO of N^84^ and the NH of V^87^. From the 10 produced families, conformer 1 (Figure 2 and Figure 3) is in agreement with all of the NOE constrains, and more specifically with the crucial interaction between NHs of N^84^ and V^87^; furthermore, it has the lowest energy (237.40 kJ mol^−1^) compared to the other families. Moreover, an H-bond was observed between the CO group of F^89^ and the final NH group of N^92^ (Figure 2).

In regard to linear MBP_82–98_ (Ala^91^) (Scheme 2B), Figure 3 presents the low-energy conformers 1 and 2, which satisfy the NOE distance constraints, and Table 3 presents the corresponding peak assignment. The low-energy conformers of linear MBP_82–98_ (Ala^91^) are shown in Figure 3. NOESY spectrum for the linear analogue presented signals only between sequential NHs; thus, no crucial NOE was observed for the conformational arrangement of the peptide (Table 3). In addition, the linear peptide was energetically minimized and subjected to molecular dynamics simulations. Ten different families were produced, from which only two (representative conformer 1 and 2) satisfied the NOE distance constraints; Table 3 presents the corresponding peak assignment. More specifically, both conformers are characterized by a β-turn (CO A^91^-NH Val94) (Figure 3). For the case of conformer 1, an H-bond was observed between the N^92^ NH and the side chain CO of N^92^. Interestingly, an H-bond between the side chain of D^82^ and the OH of T^98^ provides a more closed conformation for conformer 2, which had the lowest energy compared to all of the other families.

### 3.3. Cyclic Peptides Bind to HLA-DR2 and HLA-DR4

**Figure 4:**
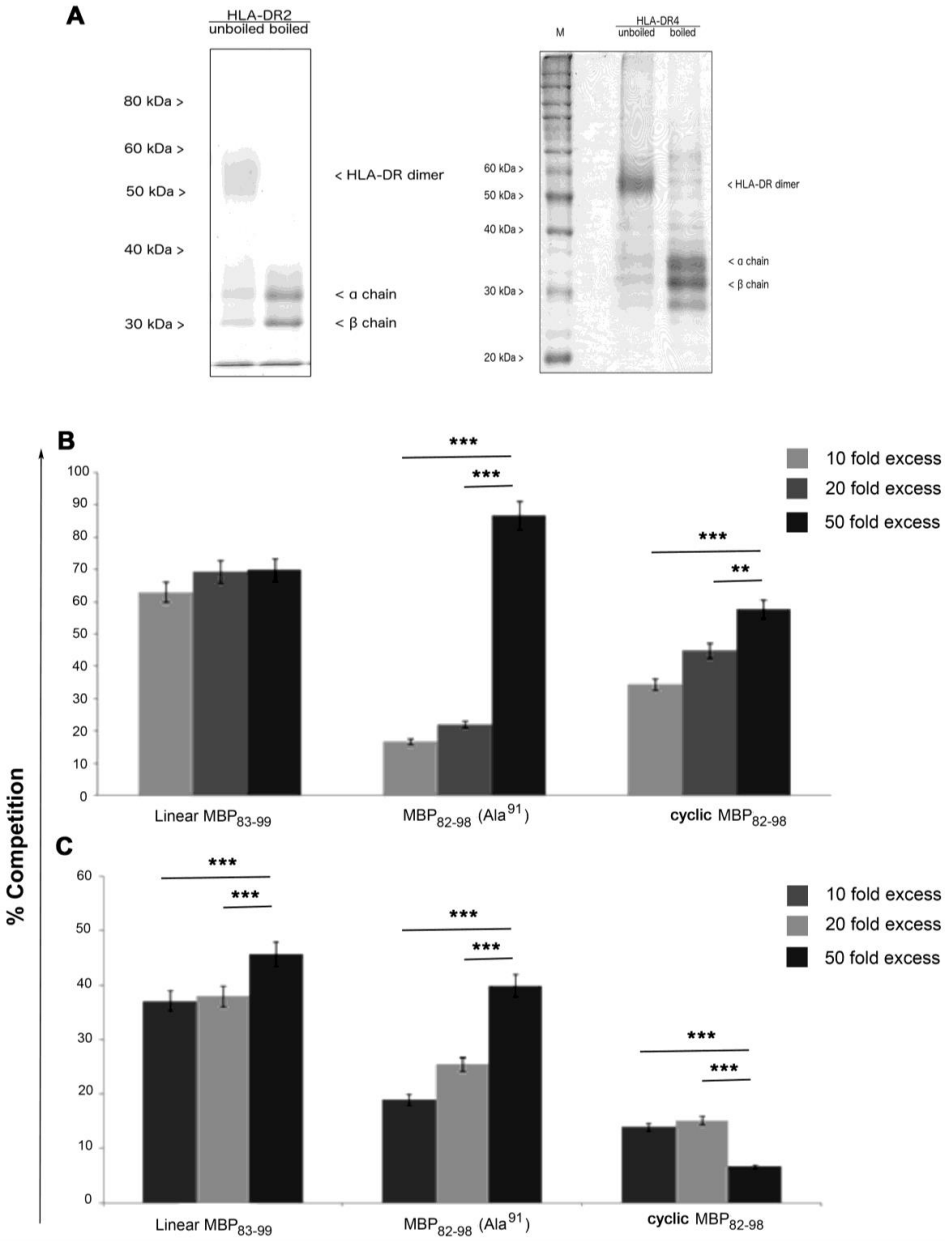
(**A**) SDS-PAGE analysis (12%) of detergent solubilized HLA-DR2 (DRB5*0101/DRB1*1501; left) or HLA-DR4 (DRB1*0401; right) where 5–8×10^9^ of the Epstein Barr virus (EBV)-transformed B cell line HTC-LAN and BSM were lysed, respectively, and their affinity was purified using the immobilized monoclonal antibody L243. After concentration on a 30-kDa membrane, 670–700 g of the human leukocyte antigen (HLA)-II were obtained and stored in phosphate buffered saline at a concentration of 0.5 mg/mL. The purity of HLA-DR2 and HLA-DR4 were about 85%. (**B**) Competition binding of the MBP_82–98_ peptide analogues in 10-fold, 20-fold, and 50-fold excess of AMCA-labeled MBP_83–99_, which is specific to the HLA-DR2b chain, was used as the peptide competitor, and (**C**) AMCA-labeled HA_306–318_, which is specific to the HLA-DR4, was used as the peptide competitor. All of the experiments were in triplicates, where ** *p* < 0.01 and *** *p* < 0.001.

HLA-DR2 and HLA-DR4 were purified in the lab, and both HLA- were shown to be pure by SDS-PAGE gels (Figure 4A). Competition binding of MBP_82–98_ peptide analogues to the HLA-DR2b chain and HLA-DR4 was determined (Figure 4). The cyclic MBP_82–98_ peptide binds to HLA-DR2 with reduced affinity compared to its linear counterpart at 10-fold, 20-fold and 50-fold excess, but still with comparable affinity (Figure 4B). The linear MBP_82–98_ (Ala^91^) peptide bound with much lower affinity, as seen in the presence of 10-fold and 20-fold excess, but was comparable to linear agonist MBP_82–98_ peptide at 50-fold excess (Figure 4B). However, the binding affinity of cyclic MBP_82–98_ peptide to HLA-DR4 was considerably lower compared to its linear counterpart and linear APL MBP_82–98_ (Ala^91^) (Figure 4C). By molecular dynamics simulations, pocket 1 (P1) in HLA-DR4 is smaller compared to pocket P1 in HLA-DR2; meanwhile, P4 in HLA-DR2 is wider, and this could be a significant factor resulting in the lower affinity of cyclic MBP_82–98_ peptide [31,32]. Nonetheless, cyclic MBP_82–98_ peptide binds to HLA-DR2 and HLA-DR4 alleles with considerable affinity; we had also shown previously that other cyclic MBP epitopes bind mouse and human MHC class II alleles [2,33]. Whether this binding affinity and its subsequent interaction with the TCR is adequate to induce anti-inflammatory effects needs to be determined.

### 3.4. Structural Elements of Different Peptides Binding to HLA Alleles

**Figure 5:**
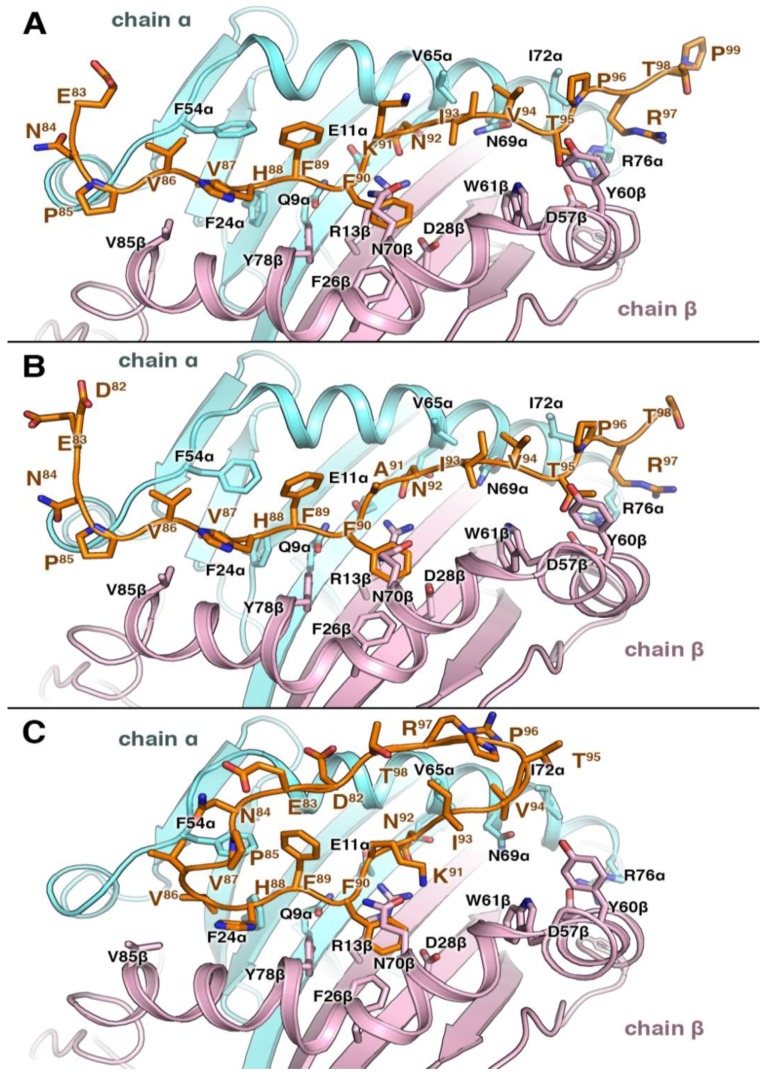
Binding of MBP_83–99_, linear MBP_82–98_ (A^91^), and cyclic MBP_82–98_ to HLA-DR2b (panels (**A**), (**B**), and (**C**), respectively). Peptides are shown in orange cartoon representation and sticks (side chains), while chains α and β are depicted in cyan and pink cartoon, respectively.

In order to evaluate the results of the binding of peptides, structural models of their binding to their corresponding HLA alleles were made. Firstly, complexes of MBP_82–98_, linear MBP_82–98_ (A^91^), and cyclic MBP_82–98_ bound to HLA-DR2b were formed using the crystal structure of MBP_83–96_ in complex with HLA-DRB1*15:01 (pdb code 1bx2) [32]. The linear MBP_82–98_ (A^91^) peptide was constructed by adding C-terminal and N-terminal residues to the MBP_83–96_ peptide, and by replacing K^91^ to A^91^. Cyclic MBP_82–98_ was constructed by joining the terminal caps. All of the complexes were then minimized to obtain the final models (Figure 5). Figure 5A shows the binding of MBP_82–98_ to the HLA-DR2b domain, where MBP residues 87–95 occupy pockets P1–P9. In Figure 5B, the A^91^ mutant is shown. Normally, the K^91^ of the MBP peptides occupies pocket P1, which is a T cell contact residue. However, since the binding assay was made in the absence of the T cell receptor, K^91^ could form a hydrogen bond with N^70^ from chain b. Therefore, the Ala^91^ mutant should have a lower affinity, as seen in Figure 4. The cyclic MBP_82–98_ peptide has a higher affinity than MBP_82–98_ (A^91^). Even though most of the HLA pockets are occupied after the cyclization (Figure 5C), no clear assumption can be made on its decreased binding affinity compared to the linear agonist peptide. However, the presence of K^91^ and a possible contact with N70 can explain the increased affinity compared to the A^91^ mutant.

**Figure 6:**
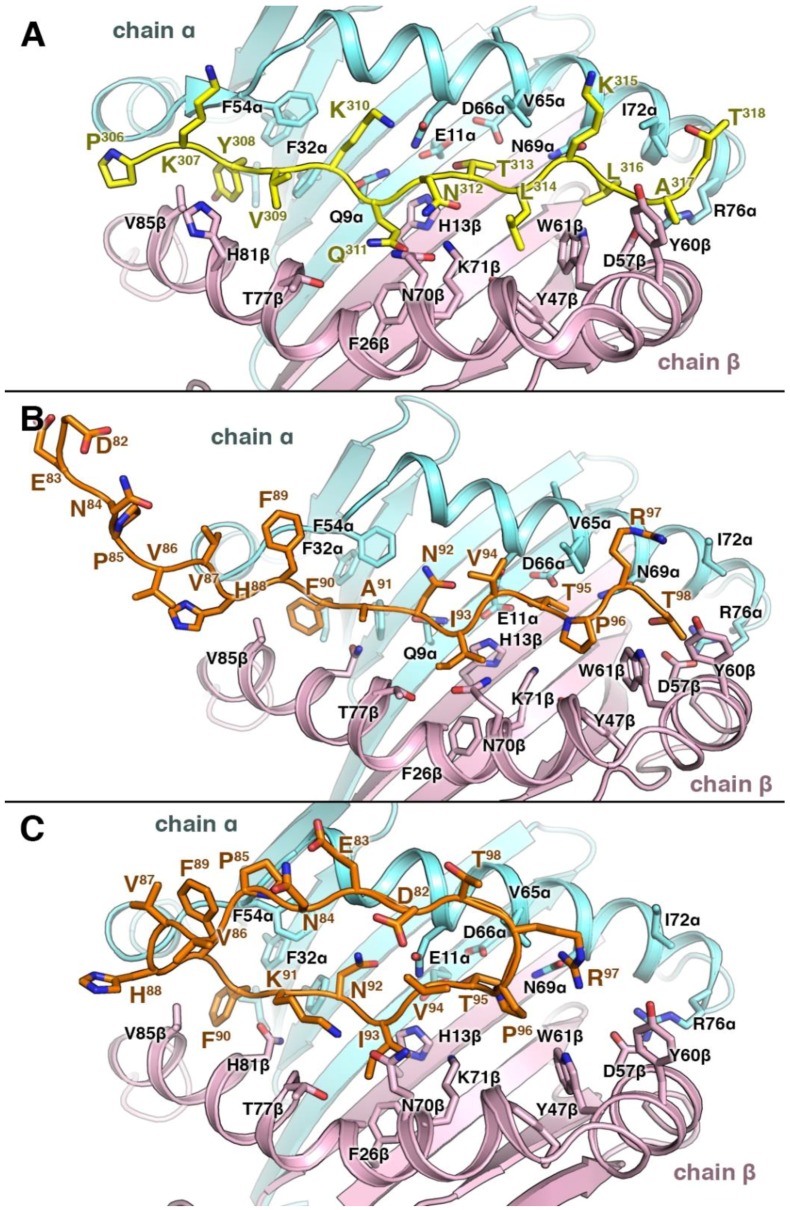
(**A**) Crystal structure of HA_306–318_ bound to HLA-DRB1*0401 (pdb code 1j8h). The peptide is shown in yellow cartoon representation and sticks (side chains), while chains α and β are depicted in cyan and pink cartoon, respectively. (**B**) Model for the binding of linear MBP_82–98_ (A^91^) and (**C**) cyclic MBP_82–98_ to HLA-DR4. MBP peptides are shown in orange cartoon representation.

For HLA-DR4, complexes of HA_306–318_, MBP_82–98_ (A^91^), and cyclic MBP_82–98_ bound to HLA-DR4 were made using the crystal structure of influenza hemagglutinin peptide (HA_306–318_) bound to HLA-DRB1*0401 (pdb code 1j8h) (Figure 6A) [34]. The docking of peptides was made on the basis of the HLA-DR2a reported crystal structure [35]. In this structure, it was noted that the MBP amino acids register shifted by three residues, compared to the HLA-DR2b crystal structures. This may be due to the presence of G^86β^ and K^71β^ in DR2β, compared to V^86β^ and A^71β^ in DR2a, resulting in a smaller P1 and a wider P4 pocket. In the case of HLA-DRB1*0401, the residues in the corresponding positions are G^86β^ and K^71β^; therefore, the binding was made according to the HLA-DR2a crystal, where pockets P1–P9 are occupied by residues F^90^–T^98^. Compared to the HLA-DR2 binding affinity experiments, in the case of HLA-DR4, MBP_82–98_ (A^91^) has a considerable higher affinity than cyclic MBP_82–98_ (Figure 4), which could be attributed to two different factors, the cyclic character and the three-residue registry shift.

## 4. Conclusions

The current study presents the synthesis of peptide analogues derived from the dirucotide peptide (MPB_82–98_) encephalitogenic sequence, the APL linear MBP_82–98_ (A^91^), and their cyclic counterparts. The peptides were synthesized in solid phase using 2-chlorotrityl-chloride resin as a solid support. The cyclization was carried out in liquid phase under stirring overnight. In our studies toward the rational design of cyclic peptides, we used structure-activity relationships (SAR) information to identify critical amino acids and applied NMR Nuclear Overhauser Effects Spectroscopy (NOESY) strategies for studying the conformation of the peptides. In particular, NMR and molecular dynamics study of linear MBP_82–98_ (A^91^) revealed a conformer 2 (Figure 3) with the head-to-tail amino acids D^82^ and T^98^ in close proximity, which was suggestive of a cyclic conformation. This NOE/MD simulations information is a lead factor for the design of head-to-tail cyclic mutants, and justified the synthesis of a cyclic dirucotide peptide. The synthesized peptides were further evaluated for their binding to HLA-DR2 and HLA-DR4 alleles with cyclic MBP_82–98_ being a strong binder with the HLA-DR2 and having lower affinity to the HLA-DR4 allele. Lastly, binding studies were performed in order to describe the binding sites when bound to HLA-DR2 and HLA-DR4 alleles. The cyclic dirucotide analogue, in addition to its APL, are promising leads to be further evaluated for their ability to modulate T cell responses in humans with MS, in particular for their ability to modulate autoimmune pro-inflammatory T cells to anti-inflammatory and regulatory T cells.

## Figures and Tables

**Table 1:** Peptide analogues and their one-letter amino acid sequence.

Peptides	Peptide Analogue of MBP_82–98_	Sequence
P1	MBP_83–99_	ENPVVHFFK^91^NIVTPRTP
P2	MBP_82–98_ (Ala^91^)	DENPVVHFFA^91^NIVTPRT
P3	cyclic MBP_82–98_	cycloDENPVVHFFK^91^NIVTPRT
P4	cyclic MBP_82–98_ (Ala^91^)	cycloDENPVVHFFA^91^NIVTPRT

MBP: myelin basic protein.

**Table 2:** Nuclear Overhauser Effects (NOE) distance constrains and corresponding distances for each class of conformers for cyclic MBP_82–98_ (A^91^).

Crucial NOEs		Distance (Å)	+10% (Å)	−10% (Å)	Conformer 237.40 kJ mol^−1^
D^82^ β′H	E^83^ β/β′H	2.22	2.44	1.99	2.62
D^82^ β′H	T^98^ γH CH3	2.92	3.21	2.62	3.21
T^95^ γH CH3	P^96^ δH	2.91	3.20	2.61	3.28
N^84^ NH	V^87^ NH	3.07	3.37	2.76	3.38
H^88^ 2,4Ar.	F^89^ NH	3.08	3.39	2.78	2.84

**Table 3:** NOE distance constraints and corresponding distances for each class of conformers for the linear MBP_82–98_ (A^91^).

Crucial NOEs		Distance (Å)	+10% (Å)	−10% (Å)	Conformer 1 248.52 kJ mol^−1^	Conformer 2 192.93 kJ mol^−1^
V^86^ NH	V^87^ NH	2.41	2.65	2.17	2.35	2.60
I^93^ NH	V^94^ NH	2.38	2.62	2.14	2.71	2.40
R^97^ NH	T^98^ NH	2.81	3.09	2.53	2.45	2.52
N^92^ NH	I^93^ NH	3.31	3.64	2.98	2.97	2.94
F^90^ NH	A^91^ NH	3.24	3.56	2.91	3.24	3.49
E^83^ NH	N^84^ NH	3.15	3.47	2.84	2.65	2.83
